# Heterosexual Men and Women Both Show a Hypothalamic Response to the Chemo-Signal Androstadienone

**DOI:** 10.1371/journal.pone.0040993

**Published:** 2012-07-16

**Authors:** Sarah M. Burke, Dick J. Veltman, Johannes Gerber, Thomas Hummel, Julie Bakker

**Affiliations:** 1 Department of Medical Psychology, VU University Medical Center, Amsterdam, The Netherlands; 2 Department of Psychiatry, VU University Medical Center, Amsterdam, The Netherlands; 3 Department of Neuroradiology, University of Dresden Medical School, Dresden, Germany; 4 Department of Otorhinolaryngology, University of Dresden Medical School, Dresden, Germany; 5 GIGA Neuroscience, University of Liege, Liege, Belgium; 6 Netherlands Institute for Neuroscience, Amsterdam, The Netherlands; Barnard College, Columbia University, United States of America

## Abstract

The odorous steroid compound 4,16-androstadien-3-one (androstadienone), found in axillary sweat, was previously reported to evoke hypothalamic activation in heterosexual women, but not in heterosexual men. However, subjects were exposed to the pure crystalline form of androstadienone, which raised the question whether the observed hypothalamic response is physiologically relevant. Therefore, in the present study, we asked whether sexually dimorphic hypothalamic responses could be measured when subjects were exposed to lower, more physiologically relevant concentrations of androstadienone. A total of 21 women and 16 men, all heterosexual, participated in our functional magnetic resonance imaging study (fMRI). Three different concentrations of androstadienone diluted in propylene glycol (10 mM “high,” 0.1 mM “medium” and 0.001 mM “low”) were delivered to the subjects’ nostrils using a computer-controlled stimulator. When exposed to the “high” androstadienone concentration, women showed stronger hypothalamic activation than men. By contrast, men showed more hypothalamic activation when exposed to the “medium” androstadienone concentrations in comparison to women. Thus, we replicated that smelling the chemo-signal androstadienone elicits a hypothalamic activation. However, this effect does not seem to be gender-specific, because androstadienone activated the hypothalamus in both men and women, suggesting that androstadienone exerts specific effects in heterosexual individuals of both sexes.

## Introduction

The importance of olfaction and social chemo-signaling in the animal kingdom is well established [1]. By contrast, the existence of chemosensory communication in humans is only beginning to be accepted. Lundström et al. [2], [3] showed that humans are able to distinguish a friend from a close relative or from a stranger, just by smelling their body odors. Furthermore, human infants, like other mammalian neonates, are attracted to odor signals secreted by the mother’s nipple and surrounding areola [4]. Both examples suggest that body odors are important in social interactions. One potential candidate for chemo-signaling in humans is the androgenic steroid 4,16-androstadien-3-one, which is found in axillary sweat [5], [6]. Several studies have suggested that androstadienone is processed in a sexually dimorphic way [7]-[10]. It has also been demonstrated that androstadienone may have gender-specific effects on the autonomic nervous system, increasing skin conductance and decreasing skin temperature in women only [11], [12]. In addition, androstadienone was found to enhance positive mood in women [7], [9], [13], [14] as well as stimulate their state of arousal, sense of being focused, and attention for socio-emotional stimuli [12], [15], [16]. Bensafi et al. [7] tested for compound concentration effects in their study on the effects of androstadienone on mood and the autonomic nervous system. Interestingly, they showed that the effects of androstadienone were only observed when androstadienone was applied in high, not physiologically relevant concentrations (6.25 mM), whereas no detectable psychological or physiological changes were apparent when androstadienone was applied in lower concentrations (0.25 mM). The absolute detection threshold for androstadienone has been reported to be 0.211 mM [17].

Studies by Savic and coworkers [18]-[20] suggested that central processing of androstadienone is sexually dimorphic, as well as specific to sexual orientation: heterosexual women and homosexual men both showed hypothalamic activation when exposed to androstadienone, which was contrasted against room air as a baseline condition. However, heterosexual men and lesbian women only showed activation in brain areas related to odor processing, such as the amygdala, piriform cortex, insula and orbito-frontal cortex. Savic et al. thus proposed that androstadienone may be a “putative male pheromone” [21] important in sexual attraction. However, these authors did not include any control odor, so that neural activation to androstadienone relative to a “higher level” could not be assessed. Moreover, subjects were exposed to the pure crystalline form of androstadienone, which raises the question whether the observed hypothalamic response is physiologically relevant, since the concentration of androstadienone excreted in sweat is so much lower [6]. Therefore, in the present study we used functional magnetic resonance imaging (fMRI) to investigate whether sexually dimorphic hypothalamic responses could be observed when subjects were exposed to lower, more physiologically relevant concentrations of androstadienone.

## Materials and Methods

### Subjects

The initial study sample consisted of 60 subjects. They were first tested for their ability to smell androstadienone in order to be able to participate in the fMRI study. We excluded 21 subjects because of anosmia to androstadienone. From the 39 remaining subjects, one did not show up for his fMRI scan and one subject was excluded from further analysis after she reported having a bisexual orientation. One of our inclusion criteria was having a heterosexual orientation which was assessed by means of self-report. Thus, finally, a total of 21 women (mean age  = 24.7, SEM  = 1.4 years) and 16 men (mean age  = 24.7, SEM  = 1.1 years) participated in the fMRI study. They were all right-handed, heterosexual volunteers and were not using any medication. Females used no oral contraceptives and all had regular menstrual cycles. Normal olfactory function of the participants was ascertained by means of the “Sniffin’ Sticks” test battery [22]. An estimation of the detection threshold for androstadienone (Steraloids Inc., Newport, RI, USA) was obtained for each participant with a three-alternative forced-choice detection test consisting of six concentrations of androstadienone dissolved in propylene glycol (10 mM, 1 mM, 0.1 mM, 0.001 mM, 0.0001 mM, 0.00001 mM), with one of the three glass jars containing androstadienone and the other two the solvent, propylene glycol (Fluka, Sigma-Aldrich Chemie Gmbh, Munich, Germany). The three glass jars, containing 10 ml of liquid each, were presented in ascending order until subjects had correctly identified androstadienone in two consecutive trials, which was then used as an estimation of the detection threshold for this participant. During the screening session, subjects were asked to report the intensity (on a scale from 0 to 10) and quality of the 10 mM, “high” concentration (on a scale from −5 to +5).

MRI sessions were planned a few days after the day of screening. Participants were asked not to wear any perfume on the day of scanning and not to eat or drink anything other than water one hour before scanning. Subjects were instructed to breathe through their mouths during the odor stimulations and females were scanned during the second or third week of their menstrual cycle, which was determined by means of self-report. Subjects gave their informed consent, according to the Declaration of Helsinki, and the study was approved by the Ethics Committee of the Medical Faculty, University of Dresden (application number EK373122009).

### Delivery of Olfactory Stimuli

Subjects were exposed to two different olfactory stimuli during the scanning sessions: 1) androstadienone and 2) 1-butanol (Merck Chemicals, Darmstadt, Germany). Androstadienone, which were diluted in propylene glycol to three different concentrations: 10 mM “high,” 0.1 mM “medium” and 0.001 mM “low,” was used to determine whether there would be gender-specific effects in hypothalamic activation as well as whether there would be any dose-dependent effects of the steroid on hypothalamic activation. The medium concentration was based on the mean detection threshold for androstadienone. Butanol was diluted in propylene glycol to a concentration of 0.01 mM and was used as a control odor. The volume of each solution used during the fMRI experiments was 20 ml. Olfactory stimuli were delivered through a tubing system to the subjects’ nostrils by means of an air-dilution olfactometer [23]. Briefly, in the olfactometer setup used in our study, air is obtained from a regular clean air outlet in the wall and directed via computer-controlled electro-pneumatic valves to 5 gas-washing bottles, which contain the liquid odorants (in our case: androstadienone in three different concentrations) and the control fluids (butanol and water). When the air enters the olfactometer, a constant airflow is achieved by using a ball-flow-meter, The air is then directed into a solenoid operated three-way pneumatic switching valve. In its turned-off-state, the air leaves the valve through the port which functions as an exhaust and maintains a steady airflow. When the airflow-valve is turned on, the air leaves this valve passing the normally open port, which is split into 5 connections. This separates the air into 5 streams and distributes it over five solenoid operated three-way pneumatic switching valves. The air is saturated with odorant as it travels through the porous frit at the bottom of the gas-washing bottles and forms small air-bubbles. Upon exiting the 5 gas washing chambers, the odorized or control air passes through 5 individual Teflon tubes to the delivery section. Just before entry into the subject’s nose, the gas-flow is reunited using t-fittings into a single flow line inside a 5 cm Teflon hose. The olfactometer uses a continuous airflow design and as a result, regardless of how many valves are simultaneously opened, the resulting airflow at the delivery unit always matches the one entering the device avoiding air-puffs at the outlet. With a total air flow of 2 L per minute, during “ON” periods every 2 seconds odors where delivered during 1 second, while during “OFF” periods subjects received odorless air. The presentation order of odors (3 × androstadienone, 1 × butanol) was randomized across subjects. After each odor session, subjects were asked to report intensity and valence of the three different androstadienone concentrations and the control odor butanol on a 10-point scale.

**Table 1:** Androstadienone threshold estimations during the screening session.

	Total (in %)	Females (n = 21)/Males (n = 16)
**10** **mM**	10.5	1/3
**1** **mM**	15.8	5/1
**0.1** **mM**	47.4	9/8
**0.01** **mM**	23.7	6/3
**0.001** **mM**	2.6	0/1

**Table 2:** Intensity ratings during screening and scan sessions on a scale from 0 to 10.

	Females (n = 21)		Males (n = 16)			
	Mean	St.dev.	Mean	St.dev.	T-value	P-value (sex difference)
**Screening**	6.9	2.3	6.4	2.2	0.64	0.526
**AND high**	4.9	2.7	3.9	2.5	1.08	0.289
**AND medium**	4.6	2.7	4.3	1.9	0.39	0.700
**AND low**	2.7	2.9	3.9	2.9	−1.27	0.214
**Butanol**	5.9	2.9	5.0	2.1	1.02	0.317

AND = androstadienone; St.dev. = standard deviation.

**Table 3:** Valence ratings during screening and scan sessions on a scale from −5 to +5.

	Females (n = 21)		Males (n = 16)			
	Mean	St.dev.	Mean	St.dev.	T-value	P-value (sex difference)
**Screening**	−1.2	2.6	−2.0	1.6	1.04	0.307
**AND high**	−0.3	1.6	−0.9	1.3	1.24	0.224
**AND medium**	−1.1	1.6	−0.5	1.6	−1.05	0.302
**AND low**	−0.3	1.6	−1.1	1.7	1.47	0.152
**Butanol**	−1.0	2.1	−1.0	1.8	0.00	1.000

AND = androstadienone; St.dev. = standard deviation.

### fMRI Protocol

Scans were performed on a 1.5 Tesla scanner (Siemens Magnetom SONATA; Siemens Healthcare, Erlangen, Germany). A gradient echo (GE) echoplanar imaging (EPI) sequence was used for functional imaging. The parameters included a 10×15 cm^2^ field of view, TR of 2240 msec, TE of 40 msec, a 90° flip angle, 27 slices, voxel resolution of 2.3×2.3×3 mm. The scanning volume was centered on the hypothalamus. Before each imaging session an automated local shimming technique was used to reduce susceptibility artifacts. A scanning session consisted of four subsessions, during each of which 108 images were acquired, lasting 4.06 min. Each odor session consisted of 6 alternating “ON-OFF” cycles over 108 scans in a classical block design. For co-registration with the functional images a T1-weighted scan was obtained (3D IR/GR sequence, TR = 2180 ms, TE = 3.93 ms).

**Figure 1:**
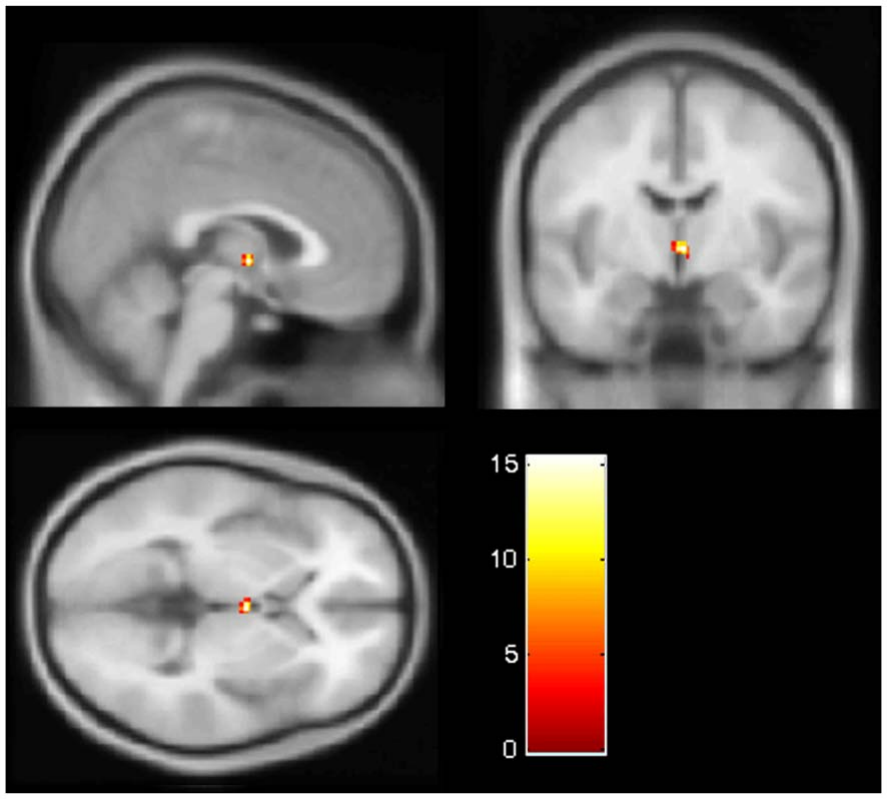
Hypothalamus activation for the interaction effect of the factors “group” and “androstadienone concentration”. F-contrast, testing for group (men and women) differences in hypothalamus activation, dependent on androstadienone concentration condition. The color bar depicts the F statistic and image coordinates (x = 2; y = −6; z = 0) are in Montreal Neurological Institute brain atlas space.

### fMRI Data Analysis

fMRI data were analyzed using SPM8 (Wellcome Trust Centre for Neuroimaging, http://www.fil.ion.ucl.ac.uk/spm). Functional images were realigned to the mean image, co-registered with the individual T1 scan and normalized to MNI (Montreal Neurological Institute) space using segmentation. Finally, images were spatially smoothed with a 4 mm full width half maximum (FWHM) isotropic Gaussian kernel. This setting was chosen not to exceed the size of the anatomical structure of interest, the hypothalamus. In order to identify the effect of odor stimulation, first level linear contrast images were entered into a general linear model (GLM), generating statistical images. These images displayed the effect of odor stimulation (“On” blocks versus “Off” blocks) in each participant. Next, these contrast images were entered into second-level group analyses to investigate the effects of group (men versus women) and odor concentration.

In order to address the main questions of the study, i.e. whether there are any sex differences in hypothalamic activation upon smelling androstadienone, and whether the hypothalamus would respond differently in the two sexes to the three androstadienone concentrations, a flexible factorial model was applied. The factors “gender,” having two levels (females and males) and “androstadienone,” having three levels (three different concentrations) were defined, as well as the factor “subject” (accounting for multiple within-subject observations), in order to investigate the interaction effects of gender and concentration. Furthermore, to correct for subjective differences in perceived odor concentrations, the behavioral variable of subjective ratings of odor intensity, obtained after each odor session, was included as a covariate in the flexible factorial model. Sex differences for each of the three androstadienone concentrations were analyzed separately using two-sample t-tests.

We defined a hypothalamic region of interest (with Marsbar, [24]) by modeling brain activation across all subjects and androstadienone concentrations, and built a region of interest for the cluster of hypothalamic activation (peak voxel MNI-coordinates: x = 2, y = −6, z = 0; volume size  = 250 mm^3^). This region of interest was applied at the second level to investigate differences between groups and concentrations. For illustrative purposes we extracted contrast values for each concentration condition of androstadienone, using this hypothalamic region of interest. It was ensured that the reported cluster fell within coordinates defined as pertaining to the hypothalamus in earlier studies [19], [20], [25]-[29]. The statistical threshold was set at p<0.05, using family-wise error (FWE) correction for multiple comparisons in small volumes.

**Figure 2:**
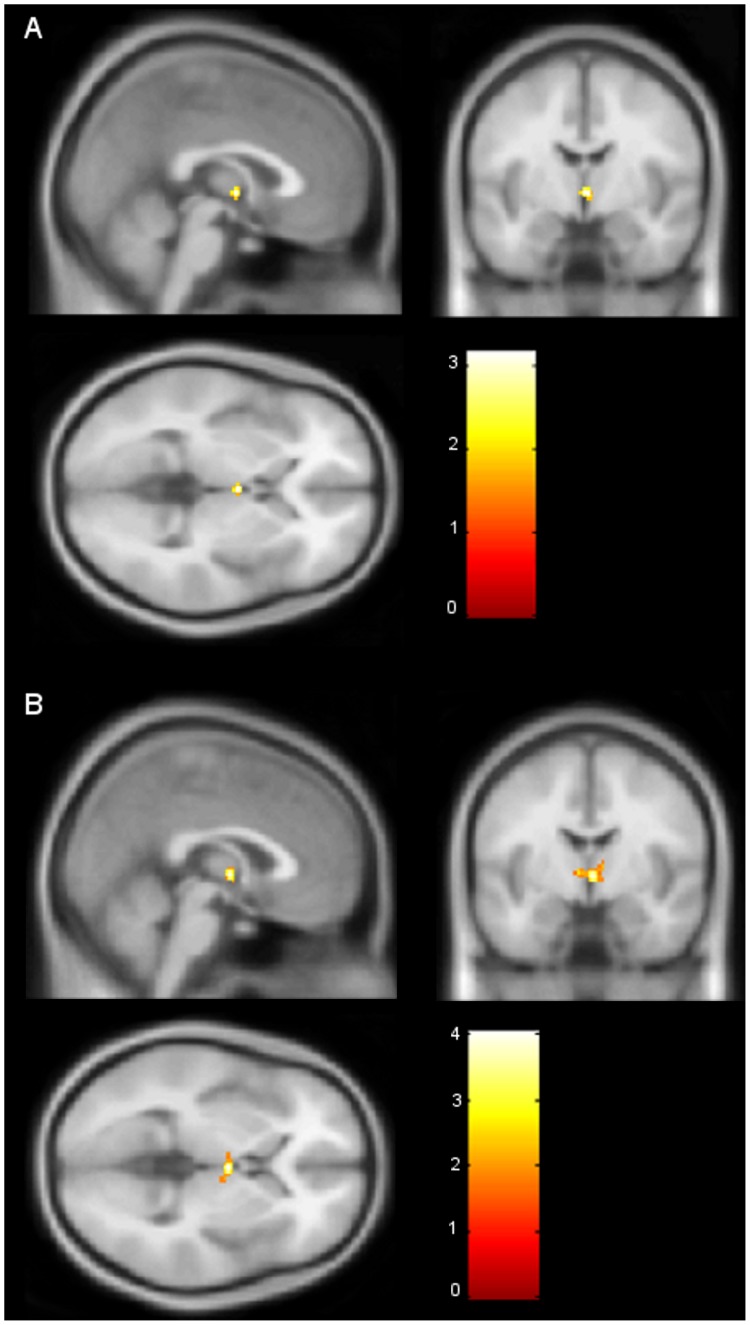
Sex differences in hypothalamus activation. (A) Hypothalamus activation in women, compared to men (directional t-contrast), who were exposed to the high concentration of androstadienone. (B) Hypothalamus activation in men, compared to women (directional t-contrast), who were exposed to the medium concentration of androstadienone. The color bar depicts the t statistic and image coordinates (x = 2; y = −6; z = 0) are in Montreal Neurological Institute brain atlas space.

**Table 4:** One-sample and two-sample t-tests, with directional t-contrasts testing for sex differences in the hypothalamus, separately for the three different androstadienone concentration conditions.

	T-value	P-value(uncorrected)	P-value(FWE-corrected)	MNI coordinatesXYZ
**Females**				
AND high	2.31	0.013	0.106	2 −6 0
AND medium	1.25	0.103	0.385	4 −6 −4
AND low	1.30	0.097	0.372	2 −8 −2
**Females > Males**				
AND high	3.39	0.001	0.013*	2 −6 0
AND medium	−1.46	0.603	0.676	4 −6 −4
AND low	1.73	0.045	0.240	2 −8 −2
**Males**				
AND high	−0.28	0.456	0.674	4 −8 −2
AND medium	4.30	0.000	0.001*	4 −6 −2
AND low	2.44	0.010	0.085	2 −4 −2
**Males > Females**				
AND high	−1.31	0.594	0.681	4 −8 0
AND medium	3.96	0.000	0.003*	2 −6 0
AND low	2.26	0.015	0.115	2 −4 0

AND = androstadienone; FWE  =  family-wise error; *  =  statistically significant at p<0.05; coordinates are in Montreal Neurological Institute (MNI) brain atlas space.

**Figure 3:**
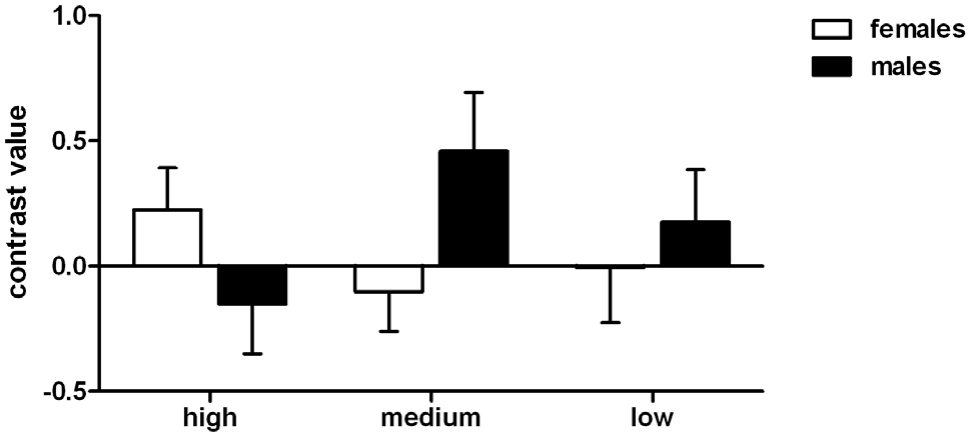
Contrast values for the three different androstadienone concentrations. Contrast values were extracted from the hypothalamus (region of interest at MNI coordinates x = 2; y = −6; z = 0, sphere diameter of 8 mm). Contrast values were determined for each of the three androstadienone concentration conditions and depicted separately for men and women. Error bars reflect the 95% confidence interval.

Additionally, we investigated a set of predefined olfactory regions of interest for possible sex differences in activation, i.e. amygdala, piriform cortex, insula, the orbito-frontal cortex (OFC) and the anterior cingulate cortex (ACC) since it has been suggested that these brain areas are implicated in the processing of androstadienone [20], [30]. For these analyses, the dataset was smoothed differently, applying a kernel of 6 mm. This setting was chosen in order to increase the signal-to-noise ratio and to account for the larger anatomical variability in cortical brain regions, compared to the (much) smaller hypothalamus. The anatomical borders for each area of interest were defined by using overlays generated by the Wake Forest University (WFU) Pickatlas [31] toolbox for SPM. Applying these regions of interest as mask images, the data were analyzed with respect to the effects of each odor (androstadienone and butanol) and for sex differences in response to smelling androstadienone (across concentrations) or butanol. The statistical threshold was set at p<0.05 (FWE corrected).

**Table 5:** Main effect analyses for the two odors androstadienone and butanol with one-sample t-tests investigating whole group brain activations in predefined olfactory regions of interest.

	L/R	T-value	P-value(uncorrected)	P-value(FWE-corrected)	MNI coordinatesx y z
**Androstadienone vs baseline**					
ACC	LR	2.682.97	0.0060.003	0.7050.488	−6 28 3014 26 24
Amygdala	LRR	4.822.621.49	0.0000.0060.070	0.002*0.2380.700	−28 2 −2230 4 −2024 −8 −12
Hypothalamus	R	3.88	0.000	0.002*	4 −6 0
Insula	LR	4.393.40	0.0000.001	0.043*0.329	−40 8 −1040 24 4
OFC	LRR	5.074.202.47	0.0000.0000.009	0.009*0.1130.980	−34 30 −644 30 −1016 56 −8
Piriform Cortex	LR	2.413.06	0.0110.002	0.3780.127	−22 4 −1830 10 −20
**Butanol versus baseline**					
ACC	LR	3.032.43	0.0020.010	0.4750.794	−10 34 242 32 24
Amygdala	LR	4.193.85	0.0000.000	0.009*0.021*	−18 −4 −1828 −4 −18
Hypothalamus	R	1.04	0.142	0.362	4 −6 −4
Insula	LR	4.974.45	0.0000.000	0.010*0.036*	−42 18 −226 22 −18
OFC	LR	4.735.75	0.0000.000	0.025*0.001*	−44 18 −224 22 −18
Piriform Cortex	LR	1.933.27	0.0300.001	0.6170.072	−26 6 −1826 8 −18
**Androstadienone versus Butanol**					
ACC	LLR	2.522.472.93	0.0080.0090.003	0.7750.8030.498	−8 2 30−12 36 −814 28 22
Amygdala	LR	1.601.22	0.0570.109	0.6140.761	−20 2 −2026 6 16
Hypothalamus	L	0.76	0.203	0.439	−2 −6 2
Insula	LR	3.453.05	0.0010.002	0.2980.531	−32 0 1238 20 −8
OFC	LR	2.893.03	0.0030.002	0.8420.778	−18 50 −1020 58 −4
Piriform Cortex	LR	1.692.34	0.0490.013	0.7170.410	−20 6 −1826 10 −16

* =  statistically significant at p<0.05; FWE  =  family-wise error; L  =  left; R  =  right; ACC  =  anterior cingulate cortex; OFC  =  orbito-frontal cortex; coordinates are in Montreal Neurological Institute (MNI) brain atlas space.

## Results

### Hedonic & Intensity Ratings

During the screening session, detection thresholds for androstadienone were estimated in all participants (Table 1) as well as their overall olfactory function by means of the Sniffin’ Sticks test battery. After each fMRI odor session the intensity and valence of the three different androstadienone concentrations and the control odor butanol were rated on a 10-point scale by the participants. There were no differences in the intensity and hedonic ratings between men and women on any of these measurements. However, overall, intensity ratings obtained during the screening session (concentration of androstadienone used: 10 mM) differed significantly (t = −3.9; df = 36; p<0.0001) from the intensity ratings obtained during scanning sessions of the high concentration of androstadienone (10 mM). Furthermore, men rated the three concentrations during the scanning sessions as evenly intense, so they did not perceive any differences between the three concentrations in the MRI (see Table 2 & 3).

### Sex & Concentration Effects – Hypothalamus

In order to determine whether the hypothalamus was responding differentially in men and women to the three concentrations of androstadienone, a flexible factorial model was applied. There was a significant interaction effect between group (men or women) and androstadienone concentration condition (F = 15.4, p<0.001; MNI-coordinates: x = 2; y = −6; z = 0), indicating that men and women show differential activation of the hypothalamus, depending on the androstadienone concentration they were exposed to during the scanning (see Figure 1). To determine whether this hypothalamic activation was modulated by subjects’ perception of the odor concentrations, intensity ratings obtained per session were added as a covariate to the model. This had no effect on the overall results.

Next, the direction (female > male or male > female) of this sex difference in the hypothalamus was tested separately for the three different androstadienone concentrations. Statistical analyses using two-sample t-tests revealed that when smelling the “high” androstadienone concentration women showed a more significant hypothalamic activation in comparison to men (T = 3.39, p = 0.013; FWE-corrected), which is in line with previous studies [20]. In contrast, when smelling the “medium” concentration, men showed a stronger hypothalamic activation than women (T = 3.96, p = 0.003; FWE-corrected). Using one-sample t-tests, males and females were also investigated separately. However, only one effect, males smelling the “medium” androstadienone concentration, survived the statistical threshold of p<0.05 (T = 4.30, p = 0.001; FWE-corrected) (see Figure 2 & Table 4). Figure 3 depicts the beta values for the sex differences across the three androstadienone concentrations.

### Androstadienone Versus Butanol

The contrast “androstadienone versus baseline” over the total sample indicated significant activations in all our olfaction-related regions of interest, except for the ACC and the piriform cortex. Contrasting “butanol versus baseline” yielded significant clusters in the amygdala, insula, the OFC, and a near-significant cluster in the piriform cortex. No significant activation could be found in the ACC and the hypothalamus. When contrasting both odors against each other, no activation in any of the regions of interest survived the statistical threshold of p<0.05, FWE-corrected (see Table 5).

Finally, we explored whether other olfactory brain regions, i.e. insula, amygdala, piriform cortex, OFC and ACC would show sex differences in activation as well. When exposed to androstadienone, regardless of concentration, none of our olfactory regions of interest showed any sex differences in activation. Also, no sexually dimorphic activation was found for the contrast “butanol versus baseline”.

## Discussion

In contrast to previous studies [20], the present study, using fMRI, showed hypothalamic activation in both men and women when exposed to androstadienone. However, sex differences were observed related to the three different concentrations of androstadienone used in the present study. When smelling the high concentration, women showed significantly more hypothalamic activation than men. By contrast, men compared to women, showed significantly stronger hypothalamic activation when smelling the medium concentration of androstadienone. Our results thus confirm that smelling androstadienone produces physiological effects in the hypothalamus in both sexes, but the direction of the sex difference depends on the concentration of androstadienone.

The female response to the high concentration of androstadienone is partially in line with the observations of Savic et al. [20] who reported that only women but not men showed hypothalamic activation when exposed to androstadienone. However, it is difficult to compare our results directly to those of Savic et al. [18], due to differences in experimental design such as odor delivery as well as applied imaging technique. In their study, 200 mg of androstadienone in crystallized, pure form was presented to the subject’s nose while being scanned using positron emission tomography. By contrast, we used an olfactometer to deliver various concentrations of androstadienone intranasally. We assume that the concentration used by Savic et al. [18] was considerably higher than our “high” condition, which is still much higher than what would occur naturally [5], [6]. Our “low” (0.001 mM) condition of androstadienone more closely approaches naturally occurring concentrations and may thus represent the most physiologically relevant condition tested here. We observed hypothalamic activation with the low concentration (see Table 4), even though only one subject was actually able to consciously detect this concentration, suggesting that androstadienone is processed without any conscious awareness of the odor.

In women, it has been shown that exposure to axillary male sweat accelerated pulses of luteinizing hormone [32], suggesting that such axillary excretions, which include androstadienone, induce neuroendocrine changes that are important for reproduction. Ferdenzi et al. [33] reported that the perceived intensity of body odors (axillary male sweat) by female raters was positively correlated with judging these odors as being more masculine. The stronger an odor, the more likely the odor was judged to be of masculine origin [34]. Furthermore, Hummel et al. [35] found that women’s hedonic ratings of androstenone varied across the menstrual cycle, peaking at ovulation. Because women are especially sensitive to strong, masculine chemo-signals around ovulation, hypothalamic activation in response to androstadienone may reflect an increase in sexual motivation [36] since our female subjects were all tested around ovulation, when endogenous estrogen levels are high. However, in the present study, we found that men also showed hypothalamic responses to androstadienone. This result is surprising in view of the claim that androstadienone may be a “putative male pheromone,” which was based on the finding that heterosexual women and homosexual men, but not heterosexual men and lesbian women, showed hypothalamic activation to androstadienone [18], [19]. However, animal studies [37] have shown that both male and female mice showed neural activation in main olfactory bulb mitral cells that project to the medial amygdala after exposure to male urinary odors, suggesting that male odors are also salient to males, perhaps related to territoriality in this species. Why would men show hypothalamic activation when smelling androstadienone? It is possible that the hypothalamic response to androstadienone reflects a general increase in drive, arousal and motivation rather than being specific to sexual behavior and motivation [38]. However, our observation of hypothalamic responses to androstadienone in heterosexual men points to a need for a more thorough analysis of possible behavioral and/or physiological actions of this compound in heterosexual men, either in the context of male-male or of male-female interactions.

Savic and coworkers [18] also showed that only brain areas related to odor processing, such as the piriform cortex and the amygdala, were activated in heterosexual men when smelling androstadienone. Female subjects, by contrast, showed no brain activation, other than the hypothalamic response when smelling androstadienone. We also investigated whether other olfactory regions were affected when exposed to androstadienone (see Table 5). In our study, both andostadienone and butanol (implemented as a non-social odor) activated the expected olfactory processing-related areas, such as the orbitofrontal cortex, insula and amygdala. It has been suggested [9], [13], [15], [39], that the steroid odor androstadienone may have neuromodulatory effects on central processes related to alertness, attention, and mood. The study by Hummer & McClintock [13] in which 50 men and women were tested in three psychological experiments, showed that androstadienone significantly enhanced attention to emotional information, but these effects were found in both sexes. Thus, brain areas other than the primary olfactory brain regions may be involved in the processing of androstadienone. Jacob et al. [30] acquired positron emission tomography scans in 10 women while smelling androstadienone, and, apart from the hypothalamus, observed changes in glucose utilization in several regions involved in mood regulation such as the amygdala and the ACC. However, since no men were included in that study, no information is available on sex-related differences. In the present study, when investigating sex differences in response to smelling androstadienone, regardless of concentration, activation in our olfactory regions of interest did not survive the statistical threshold set at 0.05, FWE-corrected. Therefore, in contrast to the study by Savic et al. [18], the hypothalamus was the only region showing a sexually dimorphic response to androstadienone.

The reported intensity of androstadienone during the screening session compared to the scanning session differed significantly, even though the presented concentration was the same for both situations (10 mM). Furthermore, intensity ratings of the three different concentration conditions during scanning were not proportional to the actual concentrations, e.g. men rated the low concentration as evenly intense as the high concentration of androstadienone. One reason for these discrepancies might simply be the different environment and conditions under which the ratings had to be made. It has been shown that a supine body position (like in an MRI scanner) in comparison to an upright body position may influence sensitivity to odors [40]-[42].

A study by Jacob et al. [13] has shown that effects of androstadienone might extend beyond immediate stimulation, thus having longer-lasting pharmacological-like effects on the organism. We cannot rule out that randomizing the different odor conditions might have had some confounding effects on brain activation or odor perception. Future studies should attempt to optimize the experimental fMRI setup to capture the cerebral activation related to processing social chemo-signals such as androstadienone.

### Conclusion

In summary, both men and women showed a hypothalamic activation upon smelling androstadienone. Thus, we were not able to replicate the earlier reported gender-specific hypothalamic activation by androstadienone. Some sex differences were observed with the three different concentrations of androstadienone used in the present study. However, the physiological relevance of these concentration-related effects remains unclear. In particular, our finding of hypothalamic responses to our “medium” concentration of androstadienone in heterosexual men points to a need for a more thorough analysis of possible behavioral and/or physiological actions of this compound in heterosexual men, either in the context of male-male or of male-female interactions.
